# Editorial: Tumor Hypoxia: Impact in Tumorigenesis, Diagnosis, Prognosis, and Therapeutics

**DOI:** 10.3389/fonc.2016.00229

**Published:** 2016-10-24

**Authors:** Christian R. Gomez

**Affiliations:** ^1^Cancer Institute, University of Mississippi Medical Center, Jackson, MS, USA; ^2^Department of Pathology, University of Mississippi Medical Center, Jackson, MS, USA; ^3^Department of Radiation Oncology, University of Mississippi Medical Center, Jackson, MS, USA

**Keywords:** hypoxia, cancer, tumorigenesis, biomarkers, therapy

**The Editorial on the Research Topic**

**Tumor Hypoxia: Impact in Tumorigenesis, Diagnosis, Prognosis, and Therapeutics**

A key to advance the rational design of better tools for cancer detection, prognosis, and therapeutics is to increase our knowledge of the tumor microenvironment. This concept is supported by a continuously growing volume of literature reviewed by Horsman and Vaupel. The stroma and tumor parenchyma coexist in a network of nutrients, growth factors, metabolites, acids, interstitial pressure, and other physiological agents (Elkhattouti et al.). The temporal and spatial dynamics of those complex interactions, considered “abnormal and adverse” relative to conditions of normal tissues (Horsman and Vaupel), affect individual cancer cells, contribute to the natural selection of distinct clones, and establish aggressive tumors.

Among the multiple components of the tumor microenvironment with strong effects on tumor aggressiveness, tumor-associated hypoxia is a particularly relevant one (Horsman and Vaupel). The abnormal and chaotic vasculature of growing solid tumors cannot provide proper delivery of oxygen to all tumor cells. Not only observed in solid tumors, but also in aggressive non-solid tumors as indicated by the Mazurier group (Deynoux et al.), irregular oxygen supply is a crucial component of tumorigenesis. A yet unexplored field related to the involvement of the hypoxia pathway in tumorigenesis is its association with heritable cancer syndromes. Thus, the inactivating germline changes in tumor-suppressor genes belonging to DNA repair pathways are associated with an increased risk of carcinogenesis. Henegan and Gomez propose the hypoxia pathway can be linked to heritable cancer syndromes. Further exploration of this hypothesis may provide emergent concepts relevant for personalized stratification and treatment of tumors associated with germline mutations of genes associated with the hypoxia pathway.

Oxygen levels affect the cells largely by regulating activity of the transcription factor hypoxia-inducible factor (HIF). Regulation of HIFα subunits is complex. As noted by Kietzmann et al., an array of kinases are key regulators of HIFα’s protein stability, subcellular localization, and transactivatory properties. It is reasonable to propose that modulation of HIFα by phosphorylation may be cell type- and cellular context-dependent. This becomes relevant when kinases can be exploited as upstream therapeutic targets of HIFα subunits in cancer therapy. HIF is also controlled by other factors. Schober and Berra pinpoint deubiquitinating enzymes, known regulators of cellular protein stability, as counterbalancing factors of ubiquitin E3 ligases (Schober and Berra). Aberrant in cancer, the ubiquitination cycle of HIFα is associated with disease progression and poor prognosis (Schober and Berra).

Other intracellular signaling mechanisms such as stress or redox response mechanisms constitute the hypoxia-activated gene expression program in tumors. For instance, the review from Mazure highlights the involvement of the voltage-dependent anion channel in cancer under the context of hypoxia. This channel, present in mitochondria, under hypoxia acts as a survival factor by promoting survival pathways in tumor cells and increased resistance to therapy agents (Mazure). These and other signaling molecules define a core for the response of cancer cells to varying oxygen conditions. Components of these networks adapt to low oxygen, play a critical role in tumor development, and offer a yet unexplored therapeutic potential.

Recently, tumor-associated hypoxia molecules have been identified as markers of poor prognosis. One example is carbonic anhydrase IX (CAIX), an enzyme involved in maintaining the cellular pH balance. Most of the studies, limited by low numbers of patients, were inconclusive. van Kuijk et al. provide the first comprehensive meta-analysis of the association between CAIX expression and treatment outcome in tumors. Patients having tumors with high CAIX expression have higher risk of locoregional failure and disease progression (van Kuijk et al.). The results of this study suggest that measuring CAIX expression can improve disease management, which in turn may decrease overtreatment and clinical relapse.

Due to the adaptation mechanisms of cancer cells to low oxygen, hypoxic tumors have reduced sensitivity to cytotoxic therapeutic agents. Hepatoma upregulated protein (HURP), a hypoxia-related molecule (1), conferred radiation resistance to prostate cancer cells (2). Noted by Espinoza et al. in their original research article, HURP expression in prostate tumors was associated with the increased expression of HIF-1α, vascular endothelial growth factor, and heat-shock protein 60, as well as increasing tumor grade. The data reinforce the relevance of hypoxia-related molecules as predictors of tumor aggressiveness, and propose a basis to further advance our understanding of the mechanistic role of hypoxia responsive molecules in therapy resistance.

On the establishment of novel approaches targeting hypoxia-related targets, the Dedhar group proposes exploiting pH regulation in the hypoxic niche (McDonald et al.). Since pH regulation is particularly relevant for cancer stem cells in hostile microenvironments, inhibition of pH regulatory enzymes, such as CAIX and monocarboxylate transporters, may result in a reduced response to hypoxia (McDonald et al.). Remarkably hypoxia promotes various mechanisms of immunoevasion (McDonald et al.). A hypothesis that will need clinical validation can be proposed: use of checkpoint inhibitors to restore antitumor immunity in combination with inhibitors of pH regulation, in the context of hypoxia, may effectively target aggressive tumors.

Equally relevant for therapeutical development is the application of novel models to study the adaptation of cancer cells to low oxygen. Chronic myeloid leukemia and hepatoblastoma are suggested by Cipolleschi et al. as tools to study low-oxygen-sensitive, -resistant, and -adapted cancer stem cells. On the basis of new findings revealed by the use of these models, application of metabolic inhibitors to target cancer stem cells may have an unexplored therapeutic potential for treatment of highly refractory disease (Cipolleschi et al.).

On the success of any experimental drug targeting tumor hypoxia, the continued adaptability of tumors to their microenvironmental stresses must be considered. Tumor cells are endowed with a very high plasticity and capacity to adapt. It is our challenge to find populations and conditions of the tumor microenvironment germane for target success. Interdisciplinary work will be the key for achievement of these goals. Benefits include; increased understanding of the involvement of hypoxia in tumorigenesis, development of better biomarker development, and more effective therapeutics.

## Author Contributions

The author confirms being the sole contributor of this work and approved it for publication.

## Conflict of Interest Statement

The author declares that the research was conducted in the absence of any commercial or financial relationships that could be construed as a potential conflict of interest.
